# Pannexin1 Stabilizes Synaptic Plasticity and Is Needed for Learning

**DOI:** 10.1371/journal.pone.0051767

**Published:** 2012-12-20

**Authors:** Nora Prochnow, Amr Abdulazim, Stefan Kurtenbach, Verena Wildförster, Galina Dvoriantchikova, Julian Hanske, Elisabeth Petrasch-Parwez, Valery I. Shestopalov, Rolf Dermietzel, Denise Manahan-Vaughan, Georg Zoidl

**Affiliations:** 1 Neuroanatomy, Medical Faculty, Ruhr-University Bochum, Bochum, Germany; 2 Cell Physiology, Faculty of Biology and Biotechnology, Ruhr-University Bochum, Bochum, Germany; 3 Neurophysiology, Medical Faculty, Ruhr-University Bochum, Bochum, Germany; 4 Ophthalmology, Bascom Palmer Eye Research Institute, University of Miami School of Medicine, Miami, Florida, United States of America; 5 Psychology, Faculty of Health, York University, Toronto, Canada; Dalhousie University, Canada

## Abstract

Pannexin 1 (Panx1) represents a class of vertebrate membrane channels, bearing significant sequence homology with the invertebrate gap junction proteins, the innexins and more distant similarities in the membrane topologies and pharmacological sensitivities with gap junction proteins of the connexin family. In the nervous system, cooperation among pannexin channels, adenosine receptors, and K_ATP_ channels modulating neuronal excitability via ATP and adenosine has been recognized, but little is known about the significance in vivo. However, the localization of Panx1 at postsynaptic sites in hippocampal neurons and astrocytes in close proximity together with the fundamental role of ATP and adenosine for CNS metabolism and cell signaling underscore the potential relevance of this channel to synaptic plasticity and higher brain functions. Here, we report increased excitability and potently enhanced early and persistent LTP responses in the CA1 region of acute slice preparations from adult Panx1^−/−^ mice. Adenosine application and N-methyl-D-aspartate receptor (NMDAR)-blocking normalized this phenotype, suggesting that absence of Panx1 causes chronic extracellular ATP/adenosine depletion, thus facilitating postsynaptic NMDAR activation. Compensatory transcriptional up-regulation of metabotropic glutamate receptor 4 (grm4) accompanies these adaptive changes. The physiological modification, promoted by loss of Panx1, led to distinct behavioral alterations, enhancing anxiety and impairing object recognition and spatial learning in Panx1^−/−^ mice. We conclude that ATP release through Panx1 channels plays a critical role in maintaining synaptic strength and plasticity in CA1 neurons of the adult hippocampus. This result provides the rationale for in-depth analysis of Panx1 function and adenosine based therapies in CNS disorders.

## Introduction

Pannexin1 (Panx1) proteins are integral membrane proteins assembling into large-conductance channels, activated by voltage, ATP, intracellular calcium, stretch, elevated extracellular potassium, or following purinergic receptor activation [1], [2], [3]. In the central nervous system (CNS) Pannexin1 is expressed in neurons and astrocytes, where it can mediate adenosine 5′-triphosphate (ATP) and glutamate release [4], [5], [6], [7]. Independent lines of evidence support a critical role of Panx1 in central nervous system (CNS) pathologies, particularly in epilepsy, stroke, or neuronal cell death [8], [9], [10], [11], [12]. In contrast, physiological functions of Panx1 *in vivo* in the adult CNS are largely uncharacterized. Panx1 is expressed in neurons and astrocytes, where it can mediate adenosine 5′-triphosphate (ATP) and glutamate release [4], [5], [6], [7], [13]. Since Panx1 is considered to be a major ATP release site, the close anatomical proximity of neurons and astroglia suggests that one of the physiological roles of Panx1 could be in synaptic feedback mechanisms initiated by ATP release. Functional crosstalk between Panx1 and purinergic receptors has been confirmed and ATP regulated ATP release shown [14], [15], [16], [17]. ATP is an agonist of the P2Y and P2X family of purinergic receptors found widely distributed in the CNS in neurons and astrocytes. Purinergic receptor activation by ATP leads to amplification of purinergic signaling thereby affecting synaptic plasticity [18]. Adenosine, a metabolic breakdown product deriving from extra- or intracellular ATP is released from both neuronal and non-neuronal sources. Both ATP and adenosine release depend on a wide variety of stimuli [19] resembling conditions know to open Panx1 channels including response to KCl depolarization, electrical stimuli or glutamate receptor activation [20]. These conditions can create sufficiently high levels of ATP to target purinergic and adenosine receptors at pre- and postsynaptic as well as extrasynaptic sites. In such circumstances modulation of neuronal activities could depend on the spatio-temporal distribution of Panx1, purinergic receptors and the stimulus thus modulating neuronal excitability, synaptic plasticity and coordination of neural networks.

The role of Panx1 and the physiological relevance of this channel need to be determined *in vivo*. The availability of knock out mice with global inactivation of Panx1 expression provides a unique opportunity to investigate a potential role in synaptic plasticity. In this study, we detected that loss of Panx1 increased excitability and potently enhanced early and persistent LTP responses in the CA1 region of acute slice preparations from adult Panx1^−/−^ mice. In line with known effects of extracellular ATP/adenosine experimental application of adenosine and N-methyl-D-aspartate receptor (NMDAR)-blocking normalized this phenotype. This suggests that lack of Panx1 disrupts a feedback mechanism involving pre- and postsynaptic activities. A compensatory transcriptional up-regulation of metabotropic glutamate receptor 4 (grm4) accompanies these adaptive changes, but has to be considered as a secondary effect. Loss of Panx1 promoted distinct behavioral changes, enhancing anxiety and impairing object recognition and spatial learning in Panx1^−/−^ mice. We conclude that ATP release through Panx1 channels plays a critical role in maintaining synaptic strength and plasticity in CA1 neurons of the adult hippocampus. This finding adds to the growing body of evidence supporting an important role of Panx1 channels in CNS physiology.

## Results and Discussion

### 

#### Genetic ablation of Panx1 alters postsynaptic responses in hippocampal CA1 area

**Figure 1:**
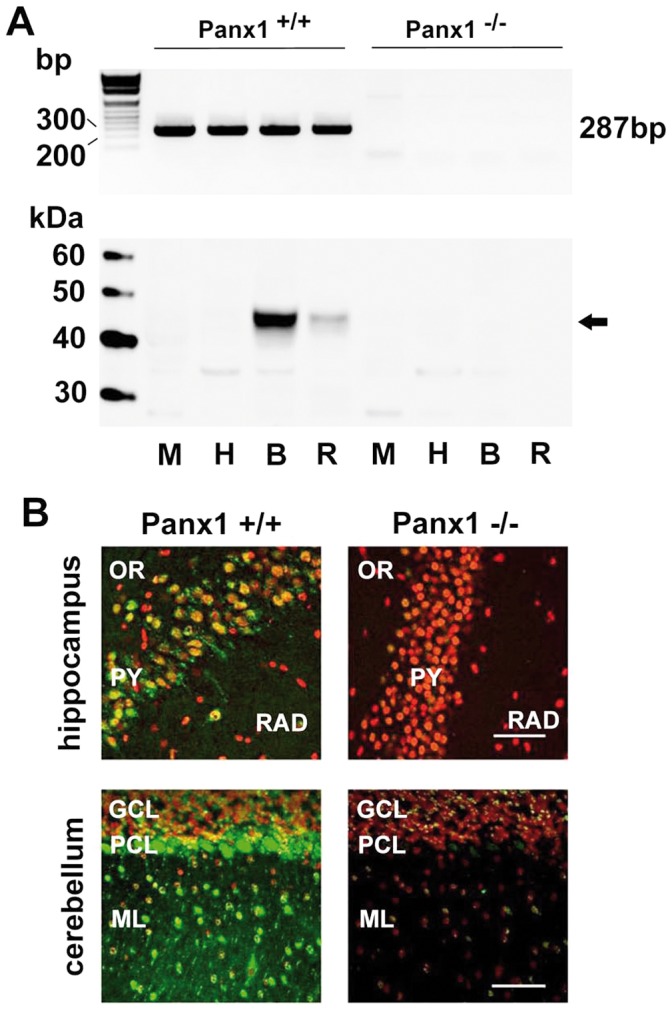
Characterization of Panx1^−/−^ mice. a, RT-PCR and western blot analysis of 4 adult Panx1^+/+^ control and Panx1^−/−^ animals. Note the lack of the 287 bp PCR amplicon representing the deleted exon4 in the Panx1 mRNA and the loss of the Panx1 protein in Panx1^−/−^ mice. b, Immunohistochemistry demonstrating loss of Panx1 protein expression in CA1 region of the hippocampus and cerebellum. Abbreviations: M, muscle, H, heart, B, brain, R, retina, OR, stratum oriens, PY, stratum pyramidale, RAD, stratum radiatum, GCL, granular cell layer, PCL, Purkinje cell layer, ML, molecular layer. Scale bars: 100 µm.

**Figure 2:**
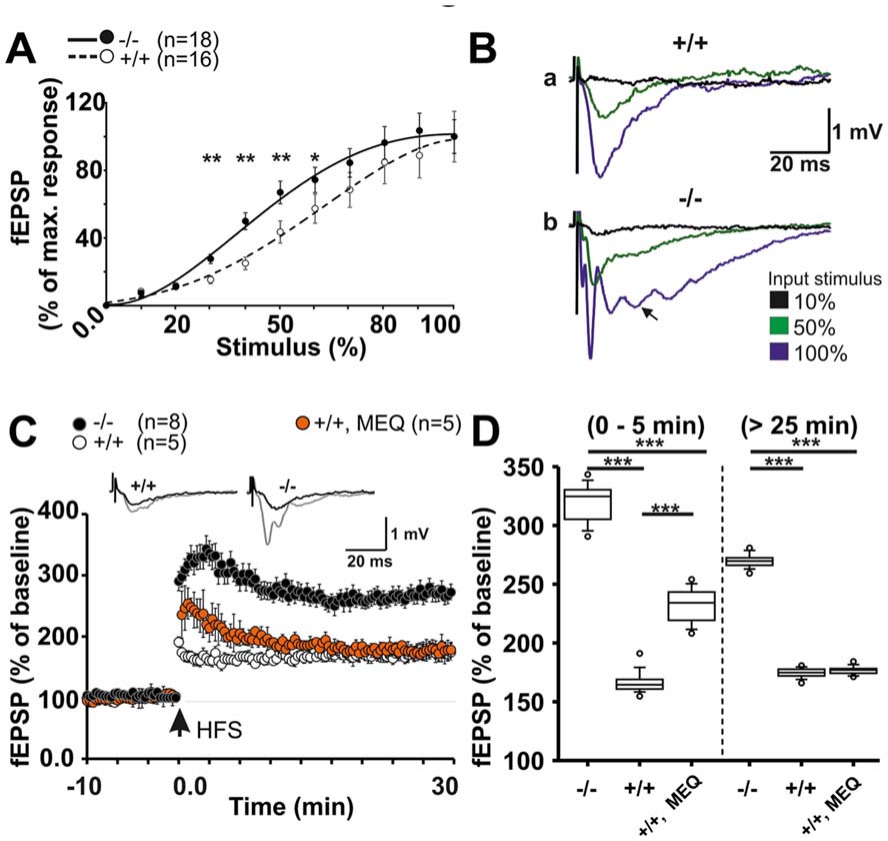
Loss of Panx1 alters postsynaptic responses in the hippocampal CA1 area. a, IO-relations demonstrate increased excitability of acute Panx1^−/−^ hippocampal slices (^−/−^, solid line; ^+/+^, dashed line). b, fEPSP sample traces at 10, 50, 100% of the maximum input stimulus intensity. At high input stimuli, a characteristic oscillatory component appears in Panx1^−/−^ but not Panx1^+/+^ mice. c, Panx1^−/−^ animals show increased LTP responses during the early (0–5 min) and persistent phase (25–30 min). Insets in (C) depict sample traces before (black) and 30 min post (grey) high frequency stimulation (HFS). Note that inhibition of Panx1 by 50 nM mefloquine (MEQ) does not fully emulate the knock out effect. c, Comparing fEPSP amplitudes reveals significant increased early and persistent LTP in Panx1^−/−^ slices. MEQ does not reconstitute the Panx1^+/+^ response to Panx1^−/−^ levels. Statistics: (a) two tailed Mann-Whitney test (non-parametric), (c) ANOVA (early: _F3,64_ = 573.7; P<0.0001; late: _F3,64_ = 259.1; P<0.0001) and Holm-Sidack post-hoc test.

Conditional knock-out (CMV-Cre/Panx1; Panx1^−/−^) with full inactivation of the *Panx1* gene in the CNS and controls of matching genetic background (Panx1/LoxP line; Panx1^+/+^), were used to test a loss-of-function condition [21]. Loss of Panx1 mRNA and protein expression was confirmed (Fig. 1*A, B*). In adult brains, no structural abnormalities were observed (Figs. S1–S3). Homozygous Panx1^−/−^ mice of two founding lines were viable and fertile, but adult animals showed indications for sensitivity to handling stress. Examination of afferent stimulus-dependent field excitatory potential (fEPSP) responses of the Schaffer-collateral CA1 synapse revealed that input-output (IO) relations in adult Panx1^−/−^derived tissues (Fig. 2*A*, black circles; n = 18) exhibited distinct responses starting from 25% of the maximum input stimulus power, with evoked responses of the KO significantly shifted towards increased excitability at 25% to 60% of the maximum input stimulus intensity (control, white circles; n = 16, P<0.001; non-parametric analysis, two tailed Mann-Whitney test). Equal sized afferent input stimulus powers (10, 50, 100%) led to distinct and enhanced fEPSP responses in Panx1^−/−^derived slices (Fig. 2*B*). Broad responses to weak input stimuli and oscillatory activity (Fig. 2*B*, arrow) at high input powers or following high-frequency stimulation were typical for Panx1^−/−^ potential traces.

High-frequency theta burst stimulation (HFS) revealed potently enhanced LTP of fEPSP amplitudes (Fig. 2*C* and insets) in Panx1^−/−^ slices (black circles) during both early (Fig. 2*C, D*; mean fEPSP: Panx1^−/−^, 320.1±3.8%; n = 8; P<0.0001; Panx1^+/+^, 216.6±2.1%; n = 5; P<0.0001, (ANOVA and Holm-Sidack post-hoc test)) and late phases (Fig. 2*D*; mean fEPSP: Panx1^−/−^, 269.8±1.4%; Panx1^+/+^, 174.6±1.0%; P<0.0001). Blocking Panx1 channels in WT slices with mefloquine (MEQ, 50 nM) [34], caused a significant increase of fEPSP responses during early (Fig. 2*C, D*; mean fEPSP: Panx1^+/+^+MEQ, 230.8±3.7%; n = 5; P<0.0001, by ANOVA), but not late-LTP, suggesting that Panx1 is important for the development of early LTP.

### Pre- and Postsynaptic Components of the Altered Panx1^−/−^ LTP

Acute pharmacological blocking of Panx1 channels only partially emulated the Panx1^−/−^ LTP-phenotype and additional physiological alterations were considered. Since Panx1 is implicated in paracrine and autocrine signaling [35], [36], initiated by activity-dependent release of ATP [14], [15], [37], [38], [39], [40], it was reasonable to speculate that lack of Panx1 in adult mice prompted an ATP misbalance, where upon intracellular postsynaptic ATP levels increase and extracellular ATP, as well as its metabolic breakdown products, fall to critically low levels.

**Figure 3:**
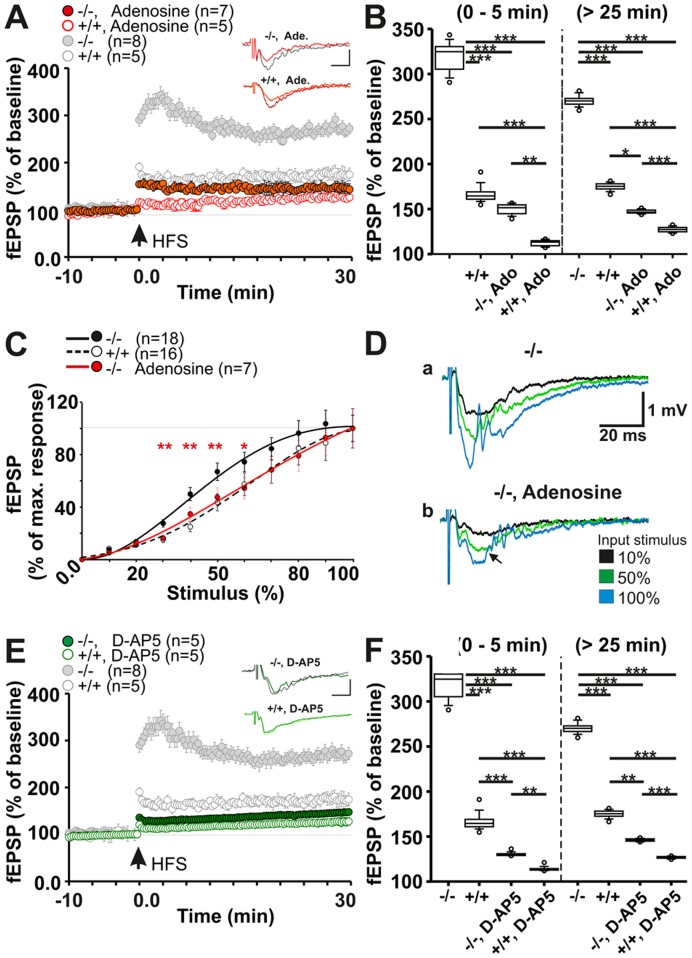
Pre- and postsynaptic components of altered Panx1^−/−^ LTP. a, Evoked LTP in presence of 3 µM adenosine: Panx1^−/−^ derived fEPSPs during early and persistent LTP phases are restored towards LTP levels of the untreated controls. Figure insets in (a) indicate ^−/−^ and ^+/+^ original responses under blocking conditions before and 30 min post HFS (scale horizontal: 10 ms, vertical: 0.5 mV). b, Comparison for early phase (0–5 min) and persistent phase LTP (25–30 min). All conditions differ significantly from the Panx1^−/−^ (see Figure 1). c, IO- correlation of the adenosine-treated Panx1^−/−^ in comparison to Panx1^−/−^ and Panx1^+/+^ under ACSF conditions. Adenosine treatment normalized the input sensitivity of the Panx1^−/−^ towards values of the untreated controls (Panx1^+/+^). d, fEPSP sample traces of the Panx1^−/−^, revealing a decrease in oscillatory activity (arrow) and amplitudes. e, NMDA dependence of ^−/−^ and ^+/+^ derived LTP after application of 50 µM D-AP5. NMDA inhibition leads to decrease of the signal during early and persistent phase of LTP, as demonstrated in (f). f, All conditions differ significantly from the Panx1^−/−^. Statistics: (b) ANOVA (early: _F3,64_ = 157.9; P<0.0001; late: _F3,64_ = 458.7; P<0.0001), (f) ANOVA (early: _F3,64_ = 184.9; P<0.0001; late: _F3,64_ = 547.4; P<0.0001).

Testing this hypothesis directly, we found that extracellular application of adenosine (3 µM) [41] almost completely normalized LTP in Panx1^−/−^ (Fig. 3*A*, filled red circles) with values falling close to levels of Panx1^+/+^ controls in ACSF (open circles). Early and late LTP responses were affected (Fig. 3A, B and Table S1; mean fEPSP early LTP: Panx1^+/+^, 112.2±0.7%; n = 7; Panx1^−/−^, 150.3±1.4%; n = 5; P<0.0001; late LTP mean fEPSP: Panx1^+/+^, 127±0.7%; Panx1^−/−^, 146.7±0.5%; P<0.001, all by ANOVA), and the oscillatory components of the fEPSP responses were abolished (Fig. 3*D*, arrow). This result is consistent with the anticonvulsant and anti-excitatory roles of adenosine, shifting the inhibitory function of A_1_ adenosine receptors towards enhanced synaptic activity by a combination of actions [42], [43].

A postsynaptic component was anticipated because of altered presynaptic excitatory neurotransmitter release. Thus, the contribution of N-methyl-D-aspartate receptors (NMDAR*)* was tested. Bath application of the NMDAR antagonist, D-AP5 (50 µM), 10 min prior to LTP recordings, significantly reduced early and persistent LTP in Panx1^−/−^ slices to levels less than those of ACSF-treated Panx1^+/+^ slices (Fig. 3*E* and Table S1; early: Panx1^+/+^, 113.8±0.5%; n = 5; Panx1^−/−^, 130.1±0.5%; n = 5; P<0.0001; late: Panx1^+/+^, 126.2±0.2%; Panx1^−/−^, 145.7±0.3%; P<0.0001, ANOVA). In general, application of D-AP5 led to significant prevention of LTP, as described for rats [44]. These results are in line with a chronic depletion of ATP/adenosine, causing sustained excitatory neurotransmitter release and increased postsynaptic excitability.

### Upregulation of Metabotropic Glutamate Receptor 4 in Panx1^−/−^ Mice

**Figure 4:**
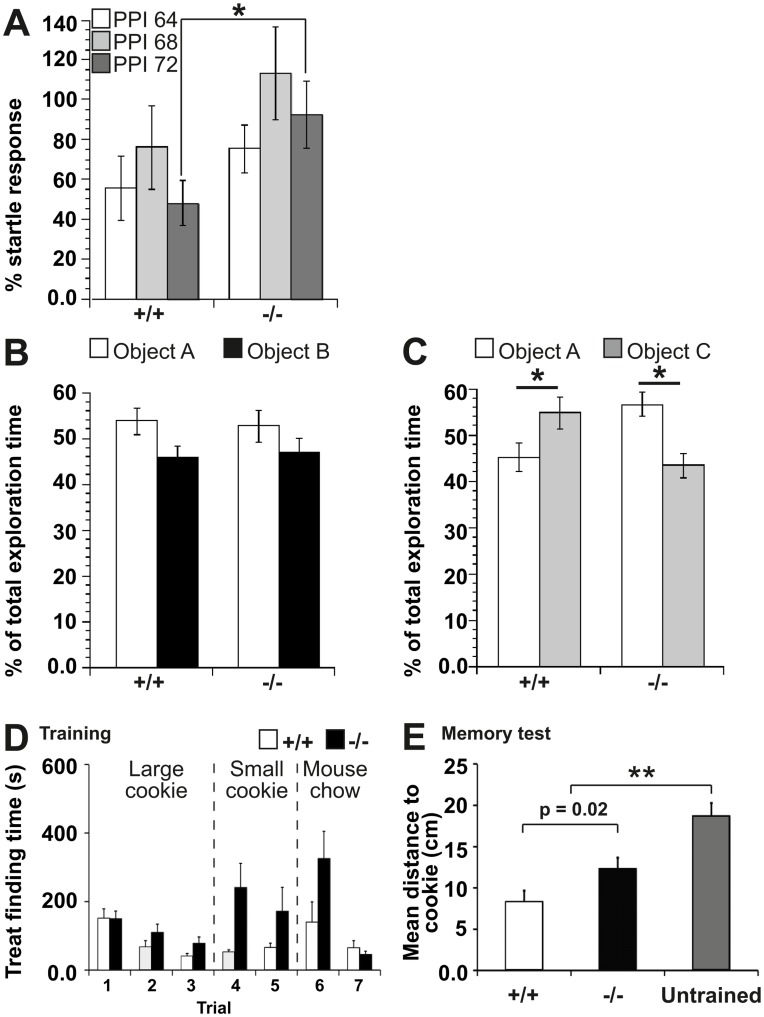
Upregulation of metabotropic glutamate receptor 4 in Panx1^−/−^ mice. a, Volcano plot representing the expression of 84 genes relevant for synaptic plasticity (Panx1^+/+^/Panx1^−/−^, n = 4). b, Inhibition of grm4 by bath application of 100 µM UBP1112. Fifteen minutes post-HFS, UBP1112 induced impaired persistent LTP in Panx1^−/−^s. Panx1^+/+^-LTP steadily increased during the entire post-HFS period without reaching the level of untreated Panx1^−/−^s. Insets in (b) indicate Panx1^−/−^ and Panx1^+/+^ original responses under blocking conditions before and 30 min post-HFS (scale horizontal: 10 ms, vertical: 0.5 mV). c, No differences were found for Panx1^−/−^s during the early LTP phase (0–5 min) compared to the ACSF treated Panx1^−/−^s. Persistent phase analysis reveals alignment of the fEPSP levels of both the UBP1112-treated ^+/+^ and ^−/−^. Statistics: (c) ANOVA (early: _F3,64_ = 637.1; P<0.0001; late: _F3,64_ = 105.9; P<0.0001).

Next, we investigated whether expression of 84 plasticity-related genes were altered in Panx1^−/−^ mice. The result was unexpected since transcriptional alterations were limited to upregulation of metabotropic glutamate receptor 4 (grm4) (Fig. 4A, and Fig. S4). All other candidates showed stable mRNA expression levels (Table S2). This transcriptional elevation was specific for adult Panx1^−/−^ mice, with no alterations found at younger ages (postnatal day 8; data not shown).

Application of the group III mGlu antagonist, UBP1112, in the grm4-sensitive dose of 100 µM [45] led to distinct changes in the LTP responses of Panx1^+/+^ (n = 6) and Panx1^−/−^ (n = 7) mice (Fig. 4B). UBP1112 elicited a significant reduction of the persistent phase of LTP in Panx1^−/−^ (blue circles) starting at 15 min post-HFS, although the decreased LTP did not reach the level of late-LTP in untreated Panx1^+/+^ controls (grey circles) (Fig. 4C; late: Panx1^+/+^ in ACSF, 174.6±0.9%; Panx1^−/−^ +UBP, 210.1±1.4%; P<0.0001, ANOVA and Holm-Sidack post-hoc test). In contrast, the antagonist enhanced LTP in Panx1^+/+^ slices. The latter finding is in line with an autoreceptor function for group III mGlu receptors [46]. Correspondingly, overexpression of grm4 may comprise an adaptive response to the enhanced excitability observed, caused by the putatively increased glutamate release.

### Behavioral Dysfunctions of Panx1^−/−^ Mice

**Figure 5:**
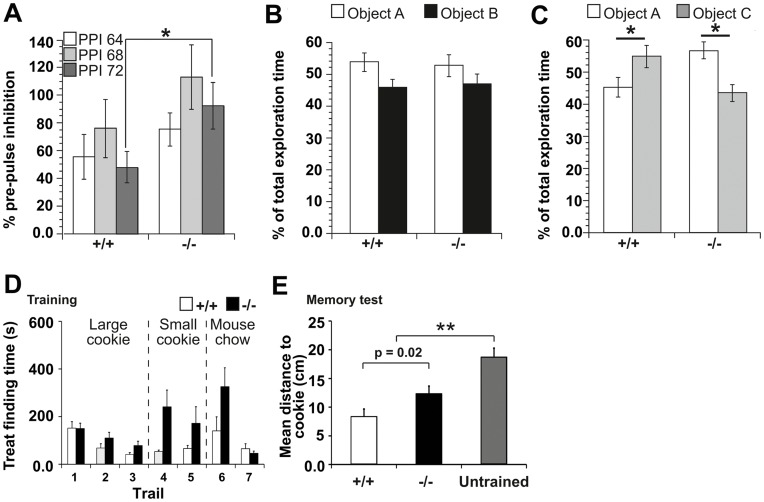
Behavioral dysfunctions of Panx1^−/−^ mice. a, Pre-pulse inhibition of the acoustic startle response (PPI) showing a tendency towards lower levels in Panx1^−/−^ (n = 8) compared to Panx1^+/+^ (n = 6) mice at the intensities of 62 and 64 dB. At the intensity of 72 dB, a statistically significant reduction of the PPI is detected. b, Object recognition was equivalent in both Panx1^+/+^ and Panx1^−/−^ mice when the animals were allowed to explored two novel objects (A and B) for 5 minutes. c, One hour later control Panx1^+/+^ mice explored the now familiar object A significantly less than the novel object C. By contrast, Panx1^−/−^ animals explored object C with significantly less intensity than object A. d, Cookie finding test: Training trials were performed on seven subsequent days, where in trial 1 and 2 a large cookie was used (500 mg), in trial 4 and 5 a smaller cookie (50 mg), and in trial 6 and 7 replaced by a very weak-odorous mouse chow. The time till the mice held the treat in their front paws is depicted in the bar diagram. Panx1^−/−^ performed equal to Panx1^+/+^ mice in this experiment (P>0.05, Student’s t-test) e, A further trial was performed after training trails where no treat was hidden (Movie S1). Bar graphs depict the mean distance of the mice to the location where the cookie was hidden during training trials for the first 60 s. Error bars represent SEM. n = 9 for each mouse group. Panx1 knock out mice have an impaired memory, as they spent less time searching at the correct position (P = 0.02). However, they still remembered the former location to some extent, as they spent significantly more time searching at the correct position as untrained animals (P<0.001, Student’s t-test).

Subsequently, the consequences of the described molecular and physiological alterations were tested at the behavioral level. Pre-pulse inhibition of the acoustic startle response was used to assess anxiety levels and sensor-motor gating capabilities. Three different pre-pulse intensities (64, 68, 72 dB) were tested in Panx1^−/−^ (n = 8) and Panx1^+/+^ mice (n = 6) [27]. An impoverished reduction in the amplitude of the startle response was evident in Panx1^−/−^ animals compared to Panx1^+/+^ controls at all intensities tested, with a statistically significant effect emerging at the intensity of 72dB (Fig. 5*A*; 64 dB: F_(1,12)_ = 1.037; P = 0.3285; 68 dB: F_(1,12)_ = 1.28; P = 0.28; 72 dB: F_(1,12)_ = 4.933; P<0.05, One-way repeated ANOVA). This result is consistent with physiological alterations in the central nervous system and, experimentally, supports a tendency to stress and anxiety-related responses observed in the Panx1^−/−^ mouse colony.

To assess cognition, Panx1^−/−^ (n = 7) and Panx1^+/+^ mice (n = 9) were tested for their ability to discriminate between a known and a new object. In the first trial, animals were exposed to two objects (A) and (B) and allowed to explore them for 5 min. One hour later, the animals were re-exposed to object A and, exposed for the first time, exposed to a new object (C). No significant difference was seen found in the exploration of object A (P = 0.986) or object B (P = 0.968) when the animal groups were compared (Fig. 5*B*). Similarly, no significant difference was evident in the level of exploration of object A versus object B for either Panx1^+/+^ or Panx1^−/−^ animals. When 1h-later, exploration of object A was compared with the novel object, C, the Panx1^+/+^ animals explored the new object significantly more than the old object (Fig. 5*C*; P<0.05). By contrast, Panx1^−/−^ mice explored the new object significantly less than the old object (P<0.05), indicating that loss of Panx1 led to deficits in object recognition memory.

Finally, we tested spatial memory capabilities by investigating the ability to remember a place where a treat was hidden during consecutive training trials. Sequential reduction of cookie size and thereby odor cues trained mice to use a memory strategy in preference to olfaction. Both groups performed equally well during initial trials with no significant differences found (n = 9 each group, p>0.05, Student’s t-test). At trial 7, both groups found the cookie showing no significant difference, suggesting that olfaction is not impaired in Panx1^−/−^ mice (Fig. 5*D*, Panx1^+/+^ = 64±21 s, Panx1^−/−^ = 45±10 s, P = 0.45). Finally, the cookie was removed to challenge the animals’ capability to remember the previously trained location. Panx1^+/+^ mice remembered significantly better where the treat was hidden (Fig. 5*E*; path-length to correct location: Panx1^+/+^, 8.4±1.3 cm; Panx1^−/−^, 12.4±1.3 cm, P = 0.02). WT mice walking moved less and spent more time searching near the correct (former) cookie location (Movie S1). However, Panx1^−/−^ mice were not memory-deficient: they were capable of remembering, to some extent the previous training trials, as their mean path-length to the former location of the cookie (Panx1^−/−^, 12.4±1.3 cm) was significantly lower compared to untrained littermates (Panx1^+/+^: 18.7±1.5 cm, P<0.001, Student’s t-test). This test suggests that Panx1^−/−^ mice are memory-impaired but not memory-deficient.

## Discussion

Recent studies have demonstrated that the lack of Panx1 improves the outcome of experimentally induced seizures in juvenile mice [47], but in adults this effect is reversed [48]. This highlights that functions of Panx1 undergo a remarkable change when the brain matures and that distinct age related roles have to be taken under consideration when exploring the physiological and pathological role(s) of Panx1. Here, our study design is based on afferent stimulus-dependent field excitatory potential (fEPSP) recordings of naïve, unchallenged tissue emphasizes the contribution of Panx1 to a defined neuronal network in adult mice. Our results demonstrate that hippocampus-dependent memory is affected in adult Panx1^−/−^ mice adding a new twist to the growing number of Panx1 functions. Intriguingly, this effect is accompanied by potently increased LTP. Saturation of LTP is related to impaired learning [49] and object recognition in mice is related to long-term depression [33]. We found that Panx1^−/−^ mice have impaired spatial memory and object recognition memory that corresponds well to a counter productive propensity towards excessive LTP. Our data support that these Panx1^−/−^mediated effects derive from an increase in neurotransmitter release and subsequent postsynaptic excitability that most likely originates from a long-term depletion in extracellular ATP as shown previously [47], which is accompanied by a compensatory upregulation of grm4 expression in our model. With Panx1 localized in strategic locations in postsynaptic terminals [25] and astrocytes [17], [35], our key findings provide compelling evidence for a role of Panx1 in synaptic physiology through signaling induced by ATP release. Our data strongly suggest that Panx1 mediates a feedback response through pre-synaptic activation of A1 receptors and inhibition of glutamate release ensuring that changes in synaptic strength remain within the dynamic range required for effective learning and memory.

## Materials and Methods

### Animals

Handling and housing of animals used in this study was performed in compliance with the German Animal Rights law and approved by the Landesamt für Natur, Umwelt und Verbraucherschutz Nordrhein-Westfalen, Germany (Permission No. 50.8735.1 Nr. 100/4). Animals were housed with a 12-hour light/dark cycle and free access to food and water. The generation of Panx1^+/+^ mice (Panx1^fl/fl^) with three LoxP consensus sequences integrated into the Panx1 gene, and knockout mice with global loss of Panx1 (Panx1^−/−^; CMV-Cre/Panx1) have been described [21]. Animals in this study were 3–9 months old.

### Reverse Transcriptase-PCR

The Message Sensor TM RT Kit was used to synthesize cDNA from total RNA obtained from dissected tissues. RT-PCR was performed with Panx1 exon4-specific primers. PCR conditions were: 95°C, 15 minutes; 40 cycles at 94°C, 30 seconds; 58°C, 30 seconds; 72°C, 1 minute. Amplified PCR products were analyzed by gel electrophoresis. Forward primer was F 5′- CCCTCTGGTCTGCTCTGTGTC-3′ and reverse primer was 5′- GGGGGTCCAGGTCCGTCTCT-3′. The exon4 specific amplicon has a size of 287 bp.

### Western Blot

Tissues were dissected, frozen instantly in liquid nitrogen, and homogenized in T-PER buffer lysis buffer (Tissue Protein Extraction Reagent by Thermo Scientific, Inc.) supplemented with complete protease inhibitor (Roche). After debris removal by low speed centrifugation and protein concentration measurements using BCA kit (Pierce), equal amounts of total protein from each sample were resolved on SDS-PAGE gradient 4–12% Bis-Tris gels and transferred to PVDF membrane (both from Invitrogen). To visualize Panx1, homogenates were extracted with 0.1% Triton X-100 and the supernatant supplemented with the loading buffer (Invitrogen). Blots were blocked in 5% milk in Tris-buffered saline (TBS, pH 7.6), probed with the primary antibody overnight, washed in 0.15% Tween20 in TBS, and incubated for 1 h with secondary antibody (1∶1,000, Amersham Biosciences, NJ, USA) diluted in TBS. Affinity purified rabbit anti-Panx1 CT-395 (Px-34) antibody was provided by Dr. D.W. Laird (University of Western Ontario, Canada) [22]. A 1∶5,000 dilution was used for western blot analysis. Anti-actin antibody was used to control protein loading. Protein bands were visualized using SuperSignal (Pierce) and quantified using the FUJIFILM software.

### RNA-expression Profiling

SABiosciences RT^2^ First Strand Kits was used for cDNA synthesis from 1 µg total RNA isolated from freshly dissected hippocampi of adult mice (n = 4 for each genotype; 6–9 month old). The kit contains an effective genomic DNA elimination step and a built-in External RNA Control for real-time PCR-based gene expression analysis with SABiosciences’ RT^2^ Profiler™ PCR Mouse Synaptic Plasticity PCR Array (all from Qiagen Inc., Toronto, Ontario M5J 2T3, Canada). All procedures were performed according to the manufacturers protocol using a DNA Engine Opticon 2 Real-Time Cycler PCR detection system (Bio-Rad Laboratories, CA, USA), with the integrated web-based software package for this PCR Array System was used for all ΔΔC_t_ based fold-change calculations from raw threshold cycle data. Data are presented as Vulcano plot, with thresholds set to: fold difference >1.5, p<0.01 (Student t-test).

### Fluorescence Immunohistochemistry

*S*ections (10 µm) from adult brain tissue of transcardially perfused mice (4% paraformaldehyde) were fixed with 3% paraformaldehyde for 15 min, permeabilized with 1% Triton X-100 for 10 min, and blocked for 1 h with 3% normal horse serum, 1% BSA in PBS, pH 7.4. A chicken anti-Panx1 antibody provided by Dr. G. Dahl (University of Miami Miller School of Medicine, Miami, FL, USA) was diluted 1∶100 [23]. Secondary antibodies were Alexa 488 nm coupled (1∶3,000). Specimen were finally stained with Hoechst 33342 (Invitrogen) and mounted with Prolong Antifade Gold (Invitrogen). Confocal image analysis was performed using the LSM 510 META system (Carl Zeiss MicroImaging GmbH,), equipped with argon and HeNe lasers, 63×(NA 1.4) oil objectives, and the LSM 510 META software as described previously [24], [25].

### Electrophysiology *in vitro*

Mice were sacrificed by cervical dislocation and horizontal hippocampal slices (350 µm) cut with a Leica VT1000 vibratome (Leica Microsystems, Wetzlar, Germany). Slices were kept in ice-cold artificial cerebrospinal fluid (ACSF) containing in mM: 124 NaCl, 2.69 KCl, 1,25 KH_2_PO_4_, 2 MgSO_4_, 10 Glucose, 2 CaCl_2_, 26 NaHCO_3_. Slices were incubated for >2 h prior to recording in ACSF at room temperature (RT) for recovery. External solutions were continuously gassed with 95% O_2_/5% CO_2_ and applied at a flow rate of 8 ml per minute. During procedures temperature was kept constant at 23±0.5°C). Field excitatory postsynaptic potentials (fEPSPs) were recorded by extracellular placement of a metal recording microelectrode (impedances 0, 5–0, 8 MΩ) (TREC-SE, Multi Channel Systems; Reutlingen, Germany) into the stratum radiatum of the hippocampal CA1 region. A concentric SNEX1200 Wolfram electrode (Hugo Sachs Elektronik, Harvard Apparatus, March-Hugstetten, Germany), was placed into the Schaffer collateral fibers to stimulate hippocampal CA1 pyramidal neurons. The fEPSP responses were driven by bipolar stimuli (50–600 µA) with a STG 1200 (Multichannel systems, Reutlingen, Germany). Before recordings were commenced, input to output correlations were determined to identify the optimum stimulation intensity, which was adjusted to 50% of the evoked maximal response amplitude. To elicit fEPSP response for long-term potentiation (LTP) recordings, 10 min of baseline stimulation at 5 Hz was performed. LTP was evoked by using high frequency stimulation (HFS) with four trains of 10 shocks at 100 Hz every 1 sec. Signals were amplified and filtered by an DAM80 extracellular amplifier (World Precision Instruments, Sarasota FL, USA), digitized at 20 kHZ and displayed, stored and analyzed using WinWCP software (Strathclyde; Biologic, Knoxville TN, USA). A 16bit analog to digital converter (BNC 2110 connected to Ni-PCi 6229; National Instruments; Munich, Germany) was used to digitize the signals.

### Pharmacology

Pharmacological agents were: Mefloquine (MEQ; Bioblocks, CA, USA), α-Methyl-3-methyl-4-phosphonophenylglycine (UBP1112; Tocris Bioscience, Ellisville, Missouri, USA), Adenosine (Sigma Aldrich, Deisenhofen, Germany), D (−)-2-Amino-5-phosphonopentanoic acid (D-AP5, Sigma Aldrich, Deisenhofen, Germany). All other chemicals were obtained from Sigma Aldrich. Pharmacology was applied 10 min before the onset of LTP recordings. Pharmacology exposure was kept stabile during the entire recording period.

### Electophysiological Data Analysis

fEPSP data were normalized as a percentage of control, based on average amplitudes from the 10 min recording immediately before LTP protocol application. For each time point, consecutive responses at 20 sec intervals were averaged and the results were expressed as the mean percentage ± standard error of the mean (s.e.m.) and summarized in table S1. LTP responses were analyzed as described [26]. All values are expressed as mean ± standard error of the mean (s.e.m.). The level of significance was set at p<0.01 [p<0.01 = *; p<0.001 = **; p<0.0001 = ***]. fEPSP amplitude data, summarized in Box- and whisker-plots in figures 1,2,3 are represented as sample minimum, lower quartile, median, sample maximum, upper quartile and considered outliers.

### Prepulse Inhibition (PPI) of the Acoustic Startle Response

All animals were subjected to 4 behavioral test sessions investigating PPI of ASR as described previously [27]. Prior to experiments animals had been individually handled and habituated to the test apparatus [28]. PPI of the ASR was measured in a sound-attenuated isolation chamber (41×41×41 cm) using a movement-sensitive piezoelectric measuring platform connected to a personal computer with an analogue to digital (AD) converter (Startle Response System, TSE, Bad Homburg, Germany) [27], [29]. During test sessions, animals were placed in a wire mesh cage (22.5 cm×8 cm×8.5 cm) mounted on the transducer-platform. For acoustic stimulation, two loudspeakers were used, mounted on both sides of the test cage at a distance of 4 cm. On the day of PPI testing, the animals were transported to the startle-box room and left undisturbed for 30 min. The experiment consisted of a 5 min acclimatization phase and a test session. During the acclimatization time, animals received background noise (60 dB sound pressure level (SPL), white noise) followed by 10 initial startle stimuli (100 dB SPL, white noise) lasting each for 20 ms (0 ms rise/fall times). The test session consisted of seven different trial types given in a pseudorandom order: (1) pulse alone (100 dB SPL white noise, 20 ms duration); (2) control (no stimulus); (3–4) pre-pulse alone (72 or 68 dB, pure tone, 10****Hz, 20 ms duration); (5–7) pre-pulse (72, 68, or 64 dB) each followed by a pulse with an inter-stimulus interval of 100 ms. A total of 10 presentations of each type was given with an inter-trial interval randomized between 20,000 ms and 30,000 ms [30]. Background noise intensity during the whole experiment was 60 dB SPL. The entire test session took about 40 min.

PPI was calculated according to the formula 100–100% 3 (PPx/PA), in which PPx is the mean acoustic startle response (ASR) of the 10 PPI trials (separate for each individual pre-pulse intensity) and PA is the mean ASR to the pulse alone trials [28]. Analysis of variance (ANOVA) was used for all comparisons (treatment, prepulse-intensity). A probability level (P) of less than 0.05 was considered statistically significant.

### Object Recognition Test

In a chamber (40×40×40 cm) that is familiar to the animals, two novel objects (i.e. A and B) were presented for 5 min. After a delay of 60 min, one familiar and one novel object (i.e. A and C) were presented to test for object recognition memory. The presentation of objects lasted for 5 min, where the animals were left to explore freely, and were removed from the recording chamber after the presentation.

The objects and the recording chambers were cleaned thoroughly between task trials to ensure the absence of olfactory cues. The objects were distinctly different from one another and heavy so that the mice could not move them. Several copies of each object were available.

Behavioral data were recorded from cameras positioned above the chambers, and digitally stored. Exploration of the objects was then analyzed *post-hoc* using the within-object area scoring system, which was defined as sniffing of the object (with nose contact or head directed to the object) within ∼2 cm radius of the object [31]. Standing, sitting or leaning on the object was not scored as object exploration. OR data were expressed as a percentage of the total exploration time for each object per experiment. [32], [33]. The results across animals were expressed in terms of mean ± s.e.m. The data were then statistically assessed using the Student’s t-test by comparing group means with the fixed value of 50%, which represents no differentiation between objects. The significance level was set at p<0.05 [32].

### Cookie Finding Test

Mice were trained in cages marked with letter “X” as visual cue. In trials 1–3 a cookie was buried beneath ∼6 cm (400 g) of woodchip bedding in their home cage. Reducing the cookie size in trials 4 and 5 reduced the olfactory component in this test. Finally, in trials 6 and 7 weakly odorous mouse chow replaced the original cookie. Training was performed on subsequent days and the treat was always hidden at the same position, with animals allowed to locate the treat in a 10 min. period. The time till the mice held the treat in their front paws was defined as finding time. After seven training trials animals were exposed to the test trial with no hidden treat. The movements were recorded and tracked with the EthoVision XT7 Software from Noldus (Wageningen, Netherland). The walking distances to the place where the cookie was hidden during the training trials were used to quantify the spatial memory of the mice. Statistical significance was tested by Student’s T-test. The significance level was set at P<0.05.

## Supporting Information

Figure S1 Morphology of adult Panx1^+/+^ and Panx1^−/−^ mouse brains. Photographs of fixed 9 month-old male control (Panx1^+/+^, left panels) and Panx1^−/−^ (right panels) brains showing no macroscopic differences in dorsal (A, B) and ventral views (C, D). Scale Bars A–D = 5 mm.
(JPG)

Figure S2 Comparison of frontal brain sections Panx1^+/+^ and Panx1^−/−^ mouse brains. (A, B) Overview representing slices (1.5 mm) at the level of the dorsal hippocampus reveals no obvious regional differences between cortex (Cx), hippocampus, thalamus, (Th), hypothalamus (Hy) and amygdala (Amy) between Panx1^+/+^ (left panels) and Panx1^−/−^ mice (right panels). All animals were 9 months old. (C–H) Toluidine-blue stained semithin sections (0.8 µm) of the hippocampus showing regions CA1 (C, D), CA3 (E, F) and dentate gyrus (DG in G, H). All regions display normal cellular and dendritic composition in both genotypes. Cp, cerebral peduncle; GR, granule cell layer, PO, polymorph layer; PY, pyramidal cell layer; RAD, stratum radiatum; SLU, stratum lucidum; Scale bars in A, B = 0.5 mm; bar in C–H = 20 µm.
(JPG)

Figure S3 Calbindin and Parvalbumin immunohistochemistry of hippocampus in Panx1^+/+^ and Panx1^−/−^ mice. (A, B) Frontal overview vibratome sections, 50 µm thick, show the characteristic calbindin staining pattern of the hippocampal subregions CA1, stratum lucidum (SLU) of CA3 with the positive mossy fibers and the strongly stained dentate gyrus (DG) in Panx1^+/+^ (left panels) and Panx1^−/−^ mice (right panels). Enlargements of the CA1 area exhibit no difference in staining of the pyramidal cell layer (PY), stratum oriens (OR) and stratum radiatum (RAD) between both genotypes. Overview micrograph of Parvalbumin immunostaining displays similar staining of Panx1^+/+^ (E) and Panx1^−/−^ mice (F). Enlargements of CA1 (G, H) show immunpositive somata of interneurons in the pyramidal cell layer and stratum oriens with the dendrites spanning all layers. Bar in A, B, E, F = 200 µm; bar in B, D, G, H = 100 µm.
(JPG)

Figure S4 Expression of selected glutamate receptor family genes. Real Time PCR was performed to analyze relative expression changes of grm1 (mGlu family I), grm2 (mGlu family II), grm4 (mGlu family III), grin1 (AMPA receptor family) and gria1 (NMDA receptor family). HSP90 and 18 sRNA expression was used for normalization.
(JPG)

Table S1 Summary of the mean ± SEM values for Panx1^+/+^ and Panx1^−/−^ derived early phase LTP and late phase LTP. Data are listed according to the bath applied pharmacological treatment. Note, that wash in of pharmacology was performed at least 10 min in advance to measurements and was kept upright during the whole phase of LTP recordings. P-Values reveal significances for Holm-Sidack post hoc comparisons, which were performed following one-way ANOVA analyses. ns = non-significant
(DOCX)

Table S2 Summary of RNA expression profiling data. Summary of data analysis using PCRArrayDataAnalysis_V3.3 software, version August 2010, (http://www.sabiosciences.com/pcrarraydataanalysis.php). Metabotropic glutamate receptor 4 (GRM4) is highlighted in red. Samples in wells H01–H05 were used for normalization of the data set.
(DOCX)

Methods S1 This section describes methods used to obtain the data described in the supporting information section.
(DOC)

Movie S1 Typical behavior of trained control (Panx1^+/+^; cage on the left) and knock-out (Panx1^−/−^; cage on the right) mice in cookie finding assay. The “X” presented in the lower left corner **signifies** where the cookie was stored during training sessions. Control mice immediately try to find the cookie in this corner, and when unsuccessful choose the top right hand corner instead. The Panx1^−/−^ explores the cage, however, lack indications of a systematic, memory**-**based exploration strategy.
(MOV)

## References

[1] MacVicar BA, Thompson RJ (2010) Non-junction functions of pannexin-1 channels. Trends in neurosciences 33: 93–102. 20022389 10.1016/j.tins.2009.11.007

[2] Penuela S, Gyenis L, Ablack A, Churko JM, Berger AC, et al.. (2012) Loss of pannexin 1 attenuates melanoma progression by reversion to a melanocytic phenotype. The Journal of biological chemistry. 10.1074/jbc.M112.377176 PMC3436541 22753409

[3] Dahl G, Keane RW (2012) Pannexin: From discovery to bedside in 11+/−4 years? Brain research. 10.1016/j.brainres.2012.04.058 PMC3590907 22771709

[4] Iwabuchi S, Kawahara K (2011) Functional significance of the negative-feedback regulation of ATP release via pannexin-1 hemichannels under ischemic stress in astrocytes. Neurochemistry international 58: 376–384. 21185900 10.1016/j.neuint.2010.12.013

[5] Orellana JA, Froger N, Ezan P, Jiang JX, Bennett MV, et al (2011) ATP and glutamate released via astroglial connexin 43 hemichannels mediate neuronal death through activation of pannexin 1 hemichannels. Journal of neurochemistry 118: 826–840. 21294731 10.1111/j.1471-4159.2011.07210.x PMC3108012

[6] Ray A, Zoidl G, Weickert S, Wahle P, Dermietzel R (2005) Site-specific and developmental expression of pannexin1 in the mouse nervous system. The European journal of neuroscience 21: 3277–3290. 16026466 10.1111/j.1460-9568.2005.04139.x

[7] Vogt A, Hormuzdi SG, Monyer H (2005) Pannexin1 and Pannexin2 expression in the developing and mature rat brain. Brain research Molecular brain research 141: 113–120. 16143426 10.1016/j.molbrainres.2005.08.002

[8] Thompson RJ, Jackson MF, Olah ME, Rungta RL, Hines DJ, et al (2008) Activation of pannexin-1 hemichannels augments aberrant bursting in the hippocampus. Science 322: 1555–1559. 19056988 10.1126/science.1165209

[9] Thompson RJ, Zhou N, MacVicar BA (2006) Ischemia opens neuronal gap junction hemichannels. Science 312: 924–927. 16690868 10.1126/science.1126241

[10] Gulbransen BD, Bashashati M, Hirota SA, Gui X, Roberts JA, et al (2012) Activation of neuronal P2X7 receptor-pannexin-1 mediates death of enteric neurons during colitis. Nature medicine 18: 600–604. 10.1038/nm.2679 PMC3321107 22426419

[11] Tymianski M (2011) Emerging mechanisms of disrupted cellular signaling in brain ischemia. Nature neuroscience 14: 1369–1373. 22030547 10.1038/nn.2951

[12] Dreier JP (2011) The role of spreading depression, spreading depolarization and spreading ischemia in neurological disease. Nature medicine 17: 439–447. 10.1038/nm.2333 21475241

[13] Garre JM, Retamal MA, Cassina P, Barbeito L, Bukauskas FF, et al (2010) FGF-1 induces ATP release from spinal astrocytes in culture and opens pannexin and connexin hemichannels. Proceedings of the National Academy of Sciences of the United States of America 107: 22659–22664. 21148774 10.1073/pnas.1013793107 PMC3012468

[14] Locovei S, Wang J, Dahl G (2006) Activation of pannexin 1 channels by ATP through P2Y receptors and by cytoplasmic calcium. FEBS letters 580: 239–244. 16364313 10.1016/j.febslet.2005.12.004

[15] Qiu F, Dahl G (2009) A permeant regulating its permeation pore: inhibition of pannexin 1 channels by ATP. American journal of physiology Cell physiology 296: C250–255. 18945939 10.1152/ajpcell.00433.2008 PMC2643853

[16] Iglesias R, Locovei S, Roque A, Alberto AP, Dahl G, et al (2008) P2X7 receptor-Pannexin1 complex: pharmacology and signaling. American journal of physiology Cell physiology 295: C752–760. 18596211 10.1152/ajpcell.00228.2008 PMC2544446

[17] Iglesias R, Dahl G, Qiu F, Spray DC, Scemes E (2009) Pannexin 1: the molecular substrate of astrocyte “hemichannels”. The Journal of neuroscience : the official journal of the Society for Neuroscience 29: 7092–7097. 19474335 10.1523/JNEUROSCI.6062-08.2009 PMC2733788

[18] Zhang JM, Wang HK, Ye CQ, Ge W, Chen Y, et al (2003) ATP released by astrocytes mediates glutamatergic activity-dependent heterosynaptic suppression. Neuron 40: 971–982. 14659095 10.1016/s0896-6273(03)00717-7

[19] Sperlagh B, Vizi ES (2011) The role of extracellular adenosine in chemical neurotransmission in the hippocampus and Basal Ganglia: pharmacological and clinical aspects. Current topics in medicinal chemistry 11: 1034–1046. 21401497 10.2174/156802611795347564 PMC3179034

[20] Buvinic S, Almarza G, Bustamante M, Casas M, Lopez J, et al (2009) ATP released by electrical stimuli elicits calcium transients and gene expression in skeletal muscle. The Journal of biological chemistry 284: 34490–34505. 19822518 10.1074/jbc.M109.057315 PMC2787310

[21] Dvoriantchikova G, Ivanov D, Barakat D, Grinberg A, Wen R, et al (2012) Genetic ablation of Pannexin1 protects retinal neurons from ischemic injury. PloS One 7: e31991. 22384122 10.1371/journal.pone.0031991 PMC3285635

[22] Penuela S, Bhalla R, Gong XQ, Cowan KN, Celetti SJ, et al (2007) Pannexin 1 and pannexin 3 are glycoproteins that exhibit many distinct characteristics from the connexin family of gap junction proteins. Journal of cell science 120: 3772–3783. 17925379 10.1242/jcs.009514

[23] Locovei S, Bao L, Dahl G (2006) Pannexin 1 in erythrocytes: function without a gap. Proceedings of the National Academy of Sciences of the United States of America 103: 7655–7659. 16682648 10.1073/pnas.0601037103 PMC1472500

[24] Kienitz MC, Bender K, Dermietzel R, Pott L, Zoidl G (2011) Pannexin 1 constitutes the large conductance cation channel of cardiac myocytes. The Journal of biological chemistry 286: 290–298. 21041301 10.1074/jbc.M110.163477 PMC3012986

[25] Zoidl G, Petrasch-Parwez E, Ray A, Meier C, Bunse S, et al (2007) Localization of the pannexin1 protein at postsynaptic sites in the cerebral cortex and hippocampus. Neuroscience 146: 9–16. 17379420 10.1016/j.neuroscience.2007.01.061

[26] Prochnow N, Gebing T, Ladage K, Krause-Finkeldey D, El Ouardi A, et al (2011) Electromagnetic field effect or simply stress? Effects of UMTS exposure on hippocampal longterm plasticity in the context of procedure related hormone release. PloS one 6: e19437. 21573218 10.1371/journal.pone.0019437 PMC3088670

[27] Manahan-Vaughan D, von Haebler D, Winter C, Juckel G, Heinemann U (2008) A single application of MK801 causes symptoms of acute psychosis, deficits in spatial memory, and impairment of synaptic plasticity in rats. Hippocampus 18: 125–134. 17924525 10.1002/hipo.20367

[28] Hohnadel E, Bouchard K, Terry AV Jr (2007) Galantamine and donepezil attenuate pharmacologically induced deficits in prepulse inhibition in rats. Neuropharmacology 52: 542–551. 17046031 10.1016/j.neuropharm.2006.08.025 PMC1913846

[29] Schneider M, Koch M (2002) The cannabinoid agonist WIN 55,212–2 reduces sensorimotor gating and recognition memory in rats. Behavioural pharmacology 13: 29–37. 11990717 10.1097/00008877-200202000-00003

[30] Schneider M, Koch M (2005) Behavioral and morphological alterations following neonatal excitotoxic lesions of the medial prefrontal cortex in rats. Experimental neurology 195: 185–198. 15935347 10.1016/j.expneurol.2005.04.014

[31] Bevins RA, Besheer J (2006) Object recognition in rats and mice: a one-trial non-matching-to-sample learning task to study ‘recognition memory’. Nature protocols 1: 1306–1311. 17406415 10.1038/nprot.2006.205

[32] Clarke JR, Cammarota M, Gruart A, Izquierdo I, Delgado-Garcia JM (2010) Plastic modifications induced by object recognition memory processing. Proc Natl Acad Sci U S A 107: 2652–2657. 20133798 10.1073/pnas.0915059107 PMC2823877

[33] Goh JJ, Manahan-Vaughan D (2012) Spatial Object Recognition Enables Endogenous LTD that Curtails LTP in the Mouse Hippocampus. Cerebral cortex. 10.1093/cercor/bhs089 PMC3615348 22510536

[34] Iglesias R, Spray DC, Scemes E (2009) Mefloquine blockade of Pannexin1 currents: resolution of a conflict. Cell communication & adhesion 16: 131–137. 20218915 10.3109/15419061003642618 PMC2854254

[35] Suadicani SO, Iglesias R, Wang J, Dahl G, Spray DC, et al (2012) ATP signaling is deficient in cultured Pannexin1-null mouse astrocytes. Glia 60: 1106–1116. 22499153 10.1002/glia.22338 PMC3348971

[36] Kawamura M Jr, Ruskin DN, Masino SA (2010) Metabolic autocrine regulation of neurons involves cooperation among pannexin hemichannels, adenosine receptors, and KATP channels. The Journal of neuroscience : the official journal of the Society for Neuroscience 30: 3886–3895. 20237259 10.1523/JNEUROSCI.0055-10.2010 PMC2872120

[37] Bao L, Locovei S, Dahl G (2004) Pannexin membrane channels are mechanosensitive conduits for ATP. FEBS letters 572: 65–68. 15304325 10.1016/j.febslet.2004.07.009

[38] Huang YJ, Maruyama Y, Dvoryanchikov G, Pereira E, Chaudhari N, et al (2007) The role of pannexin 1 hemichannels in ATP release and cell-cell communication in mouse taste buds. Proceedings of the National Academy of Sciences of the United States of America 104: 6436–6441. 17389364 10.1073/pnas.0611280104 PMC1851090

[39] Schenk U, Westendorf AM, Radaelli E, Casati A, Ferro M, et al (2008) Purinergic control of T cell activation by ATP released through pannexin-1 hemichannels. Science signaling 1: ra6. 18827222 10.1126/scisignal.1160583

[40] Chekeni FB, Elliott MR, Sandilos JK, Walk SF, Kinchen JM, et al (2010) Pannexin 1 channels mediate ‘find-me’ signal release and membrane permeability during apoptosis. Nature 467: 863–867. 20944749 10.1038/nature09413 PMC3006164

[41] Hamil NE, Cock HR, Walker MC (2012) Acute down-regulation of adenosine A(1) receptor activity in status epilepticus. Epilepsia 53: 177–188. 22150479 10.1111/j.1528-1167.2011.03340.x

[42] Mohammad-Zadeh M, Mirnajafi-Zadeh J, Fathollahi Y, Javan M, Jahanshahi A, et al (2009) The role of adenosine A(1) receptors in mediating the inhibitory effects of low frequency stimulation of perforant path on kindling acquisition in rats. Neuroscience 158: 1632–1643. 19041928 10.1016/j.neuroscience.2008.11.008

[43] Vianna EP, Ferreira AT, Dona F, Cavalheiro EA, da Silva Fernandes MJ (2005) Modulation of seizures and synaptic plasticity by adenosinergic receptors in an experimental model of temporal lobe epilepsy induced by pilocarpine in rats. Epilepsia 46 Suppl 5166–173. 10.1111/j.1528-1167.2005.01027.x 15987273

[44] Grover LM, Kim E, Cooke JD, Holmes WR (2009) LTP in hippocampal area CA1 is induced by burst stimulation over a broad frequency range centered around delta. Learning & memory 16: 69–81. 19144965 10.1101/lm.1179109 PMC2632851

[45] Conn PJ, Pin JP (1997) Pharmacology and functions of metabotropic glutamate receptors. Annual review of pharmacology and toxicology 37: 205–237. 10.1146/annurev.pharmtox.37.1.205 9131252

[46] Gereau RWt, Conn PJ (1995) Multiple presynaptic metabotropic glutamate receptors modulate excitatory and inhibitory synaptic transmission in hippocampal area CA1. The Journal of neuroscience : the official journal of the Society for Neuroscience 15: 6879–6889. 7472445 10.1523/JNEUROSCI.15-10-06879.1995 PMC6578030

[47] Santiago MF, Veliskova J, Patel NK, Lutz SE, Caille D, et al (2011) Targeting pannexin1 improves seizure outcome. PloS One 6: e25178. 21949881 10.1371/journal.pone.0025178 PMC3175002

[48] Kim JE, Kang TC (2011) The P2X7 receptor-pannexin-1 complex decreases muscarinic acetylcholine receptor-mediated seizure susceptibility in mice. The Journal of clinical investigation 121: 2037–2047. 21505260 10.1172/JCI44818 PMC3083785

[49] Moser EI, Krobert KA, Moser MB, Morris RG (1998) Impaired spatial learning after saturation of long-term potentiation. Science 281: 2038–2042. 9748165 10.1126/science.281.5385.2038

