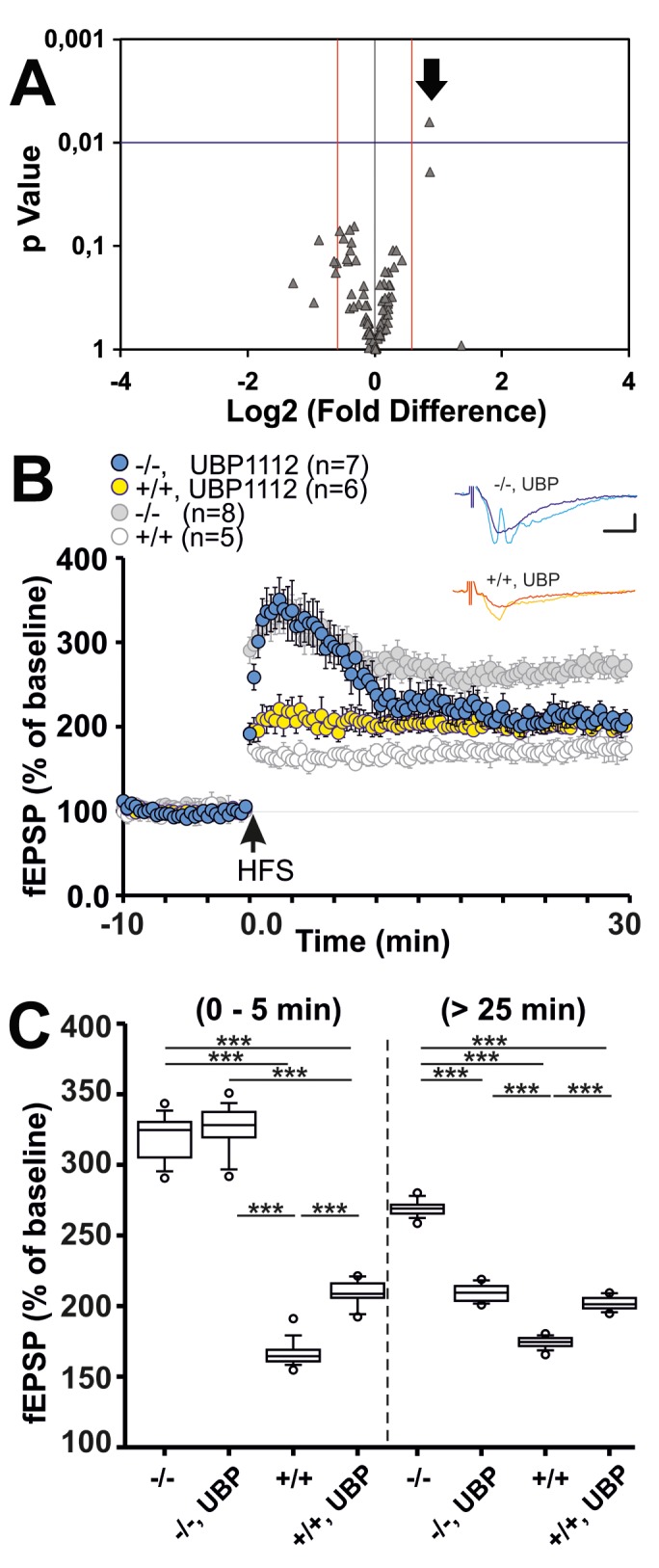# Correction: Pannexin1 Stabilizes Synaptic Plasticity and Is Needed for Learning

**DOI:** 10.1371/annotation/d0972416-5fef-4f89-9c09-cb4cb0c6295d

**Published:** 2013-01-14

**Authors:** Nora Prochnow, Amr Abdulazim, Stefan Kurtenbach, Verena Wildförster, Galina Dvoriantchikova, Julian Hanske, Elisabeth Petrasch-Parwez, Valery I. Shestopalov, Rolf Dermietzel, Denise Manahan-Vaughan, Georg Zoidl

The image for Figure 4 is incorrect. Please see the correct image for Figure 4 here: 

**Figure:**