# Supplementary figures and images for: Pannexin1 Stabilizes Synaptic Plasticity and Is Needed for Learning
Source: PLoS One. 2012 Dec 20;7(12):e51767. doi: 10.1371/journal.pone.0051767 (PMC3527502; doi:10.1371/journal.pone.0051767)

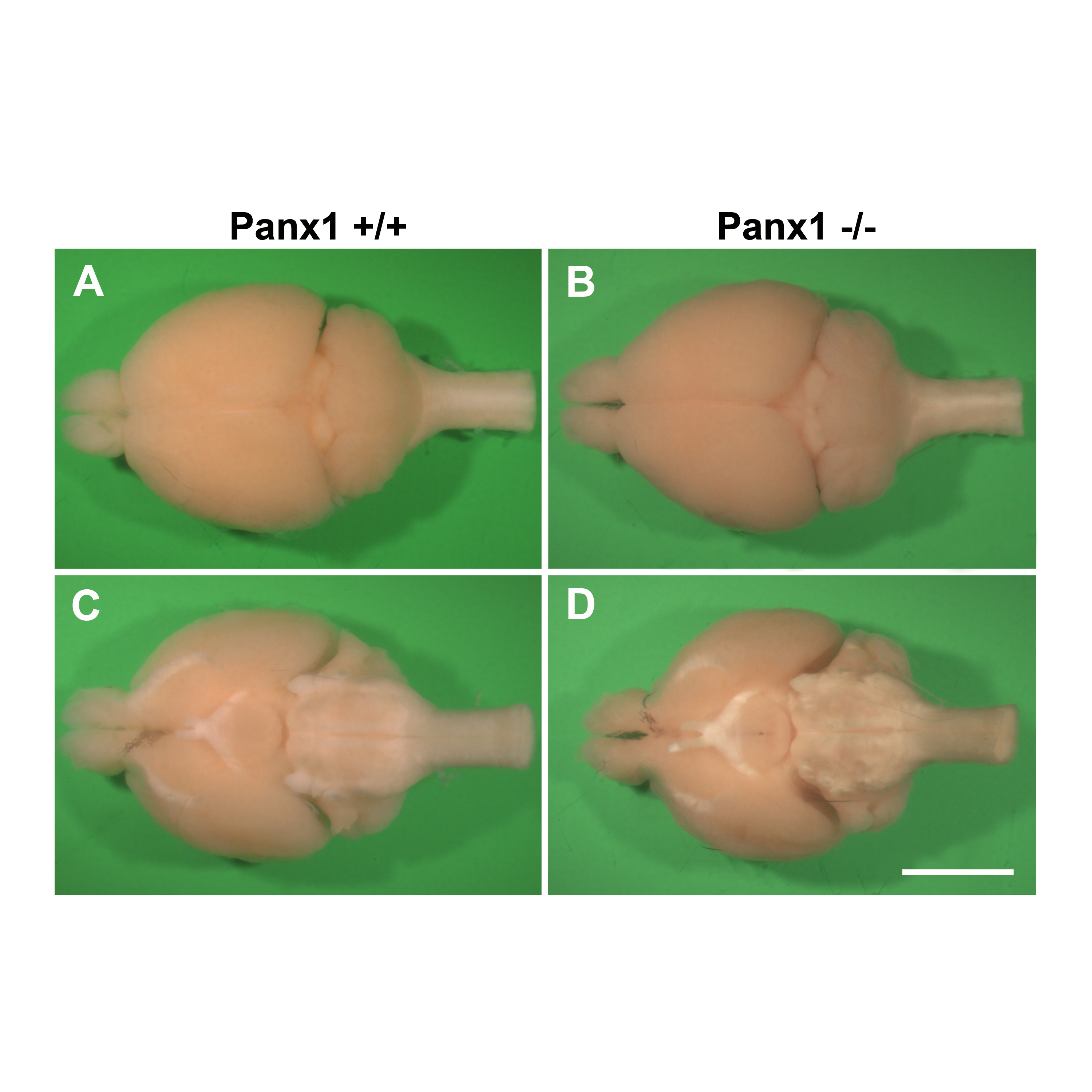

Supplement: Figure S1 — Morphology of adult Panx1+/+ and Panx1−/− mouse brains. Photographs of fixed 9 month-old male control (Panx1+/+, left panels) and Panx1−/− (right panels) brains showing no macroscopic differences in dorsal (A, B) and ventral views (C, D). Scale Bars A–D = 5 mm. (JPG) [file pone.0051767.s001.jpg]

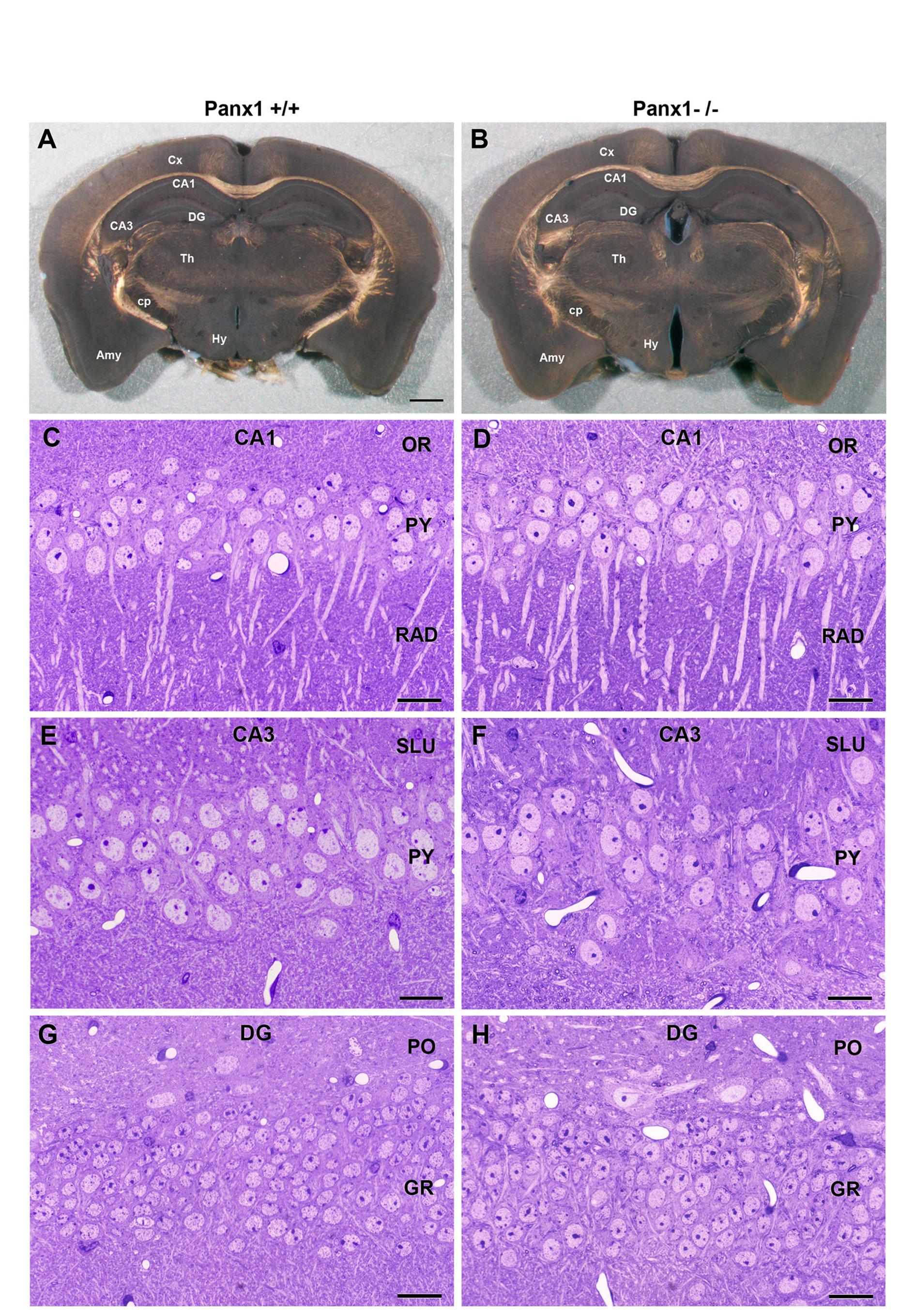

Supplement: Figure S2 — Comparison of frontal brain sections Panx1+/+ and Panx1−/− mouse brains. (A, B) Overview representing slices (1.5 mm) at the level of the dorsal hippocampus reveals no obvious regional differences between cortex (Cx), hippocampus, thalamus, (Th), hypothalamus (Hy) and amygdala (Amy) between Panx1+/+ (left panels) and Panx1−/− mice (right panels). All animals were 9 months old. (C–H) Toluidine-blue stained semithin sections (0.8 µm) of the hippocampus showing regions CA1 (C, D), CA3 (E, F) and dentate gyrus (DG in G, H). All regions display normal cellular and dendritic composition in both genotypes. Cp, cerebral peduncle; GR, granule cell layer, PO, polymorph layer; PY, pyramidal cell layer; RAD, stratum radiatum; SLU, stratum lucidum; Scale bars in A, B = 0.5 mm; bar in C–H = 20 µm. (JPG) [file pone.0051767.s002.jpg]

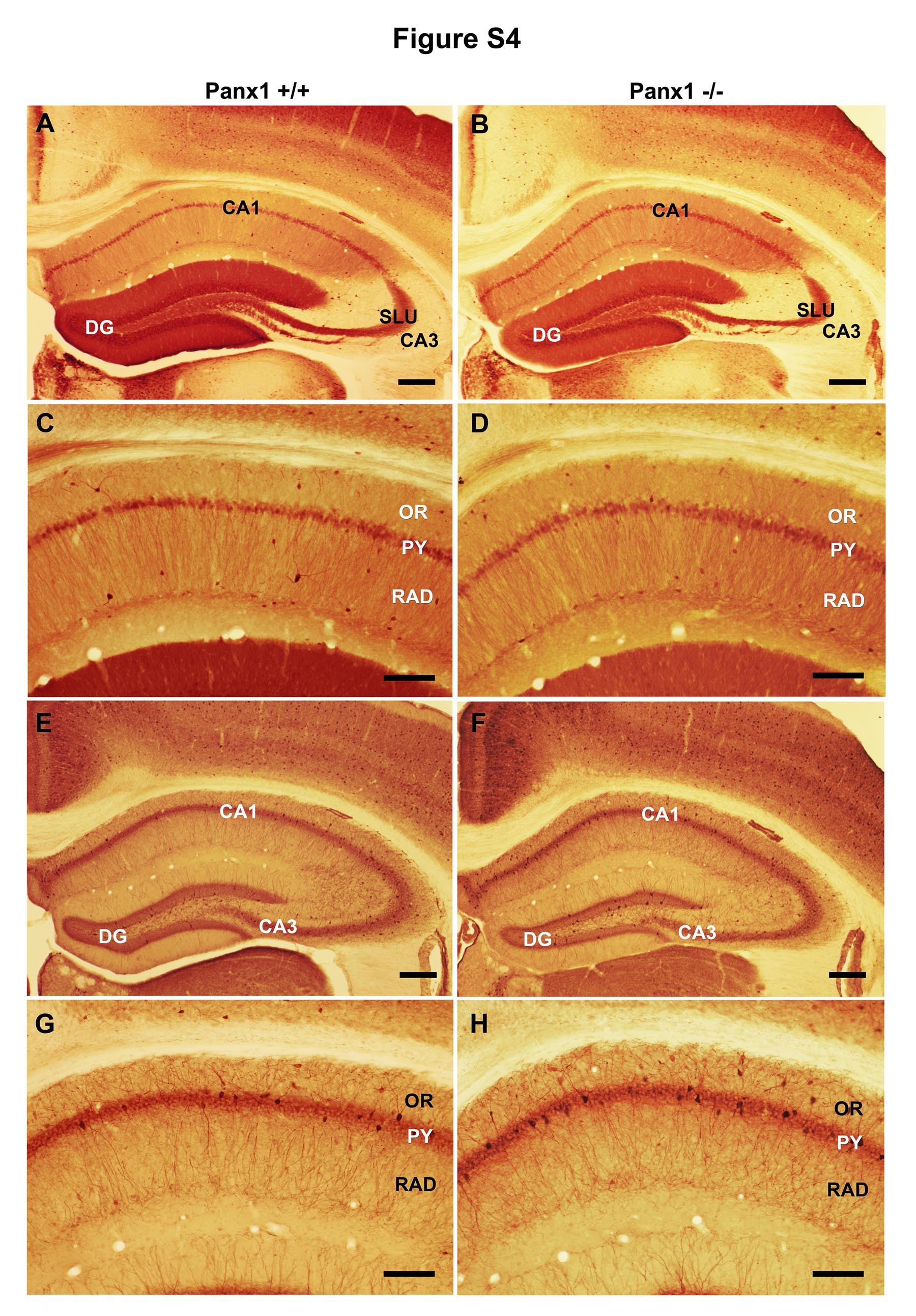

Supplement: Figure S3 — Calbindin and Parvalbumin immunohistochemistry of hippocampus in Panx1+/+ and Panx1−/− mice. (A, B) Frontal overview vibratome sections, 50 µm thick, show the characteristic calbindin staining pattern of the hippocampal subregions CA1, stratum lucidum (SLU) of CA3 with the positive mossy fibers and the strongly stained dentate gyrus (DG) in Panx1+/+ (left panels) and Panx1−/− mice (right panels). Enlargements of the CA1 area exhibit no difference in staining of the pyramidal cell layer (PY), stratum oriens (OR) and stratum radiatum (RAD) between both genotypes. Overview micrograph of Parvalbumin immunostaining displays similar staining of Panx1+/+ (E) and Panx1−/− mice (F). Enlargements of CA1 (G, H) show immunpositive somata of interneurons in the pyramidal cell layer and stratum oriens with the dendrites spanning all layers. Bar in A, B, E, F = 200 µm; bar in B, D, G, H = 100 µm. (JPG) [file pone.0051767.s003.jpg]

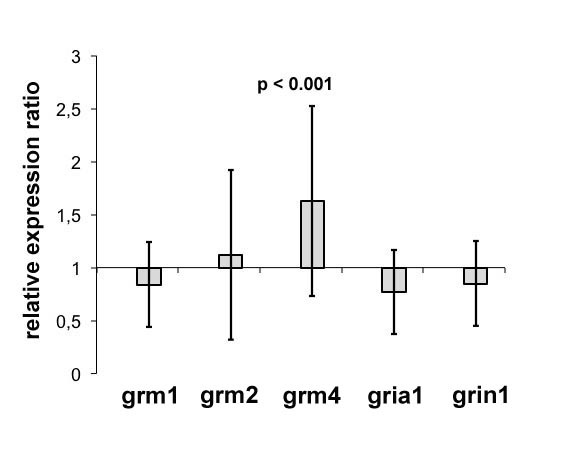

Supplement: Figure S4 — Expression of selected glutamate receptor family genes. Real Time PCR was performed to analyze relative expression changes of grm1 (mGlu family I), grm2 (mGlu family II), grm4 (mGlu family III), grin1 (AMPA receptor family) and gria1 (NMDA receptor family). HSP90 and 18 sRNA expression was used for normalization. (JPG) [file pone.0051767.s004.jpg]
