# Supplementary material for: Pannexin1 Stabilizes Synaptic Plasticity and Is Needed for Learning
Source: PLoS One. 2012 Dec 20;7(12):e51767. doi: 10.1371/journal.pone.0051767 (PMC3527502; doi:10.1371/journal.pone.0051767)
Supplement: Table S1 — Summary of the mean ± SEM values for Panx1+/+ and Panx1−/− derived early phase LTP and late phase LTP. Data are listed according to the bath applied pharmacological treatment. Note, that wash in of pharmacology was performed at least 10 min in advance to measurements and was kept upright during the whole phase of LTP recordings. P-Values reveal significances for Holm-Sidack post hoc comparisons, which were performed following one-way ANOVA analyses. ns = non-significant (DOCX) [file pone.0051767.s005.docx]

**Supplementary Table 1**

|  | **Bath application** | **WT** | **KO** | **p _WT vs. KO_** |
| --- | --- | --- | --- | --- |
| **Early** | ACSF | 166.3 ± 2.1; (n = 5) | 320.1 ± 3.8; (n = 8) | P < 0.0001 |
|  | Mefloquine [50 nM] | 230.8 ± 3.7; (n = 5) | 315.2 ± 5.1; (n = 6) | P < 0.0001 |
|  | Adenosine [30 µM] | 112.2 ± 0.7; (n = 5) | 150.3 ±1.4; (n = 7) | P < 0.001 |
|  | D-AP5 [50 µM] | 113.8 ± 0.6; (n = 5) | 130.1 ± 0.5; (n = 5) | P < 0.001 |
|  | UBP1112 [100 µM] | 208.6 ± 2.2; (n = 6) | 325.5 ± 4.1; (n = 7) | P < 0.0001 |
|  |  |  |  |  |
| **Late** | ACSF | 174.6 ± 0.9; (n = 5) | 269.8 ± 1.3; (n = 8) | P < 0.0001 |
|  | Mefloquine [50 nM] | 176.6 ± 0.8; (n = 5) | 257.0 ± 2.5; (n = 6) | P < 0.0001 |
|  | Adenosine [30 µM] | 127.0 ± 0.7; (n = 5) | 146.7 ± 0.5; (n = 7) | P < 0.0001 |
|  | D-AP5 [50 µM] | 126.2 ± 0.3; (n = 5) | 145.7 ± 0.4; (n = 5) | P < 0.0001 |
|  | UBP1112 [100 µM] | 202.3 ± 1.1; (n = 6) | 210.1 ± 0.9; (n = 7) | P = 0.49, ns |

**Supplementary Table 1** The table contains the mean ± SEM values for Panx1 +/+ and Panx1 -/- derived early phase LTP (fEPSP amplitudes as percentage of baseline during the first 5 min post HFS) and late phase LTP (fEPSP amplitudes as percentage of baseline during 25^th^ and 30^th^ min post HFS). Data are listed according to the bath applied pharmacological treatment. Note, that wash in of pharmacology was performed at least 10 min in advance to measurements and was kept upright during the whole phase of LTP recordings. P-Values reveal significances for Holm-Sidack post hoc comparisons, which were performed following one-way ANOVA analyses. ns = non-significant
