# Supplementary material for: Pannexin1 Stabilizes Synaptic Plasticity and Is Needed for Learning
Source: PLoS One. 2012 Dec 20;7(12):e51767. doi: 10.1371/journal.pone.0051767 (PMC3527502; doi:10.1371/journal.pone.0051767)
Supplement: Table S2 — Summary of RNA expression profiling data. Summary of data analysis using PCRArrayDataAnalysis_V3.3 software, version August 2010, (http://www.sabiosciences.com/pcrarraydataanalysis.php). Metabotropic glutamate receptor 4 (GRM4) is highlighted in red. Samples in wells H01–H05 were used for normalization of the data set. (DOCX) [file pone.0051767.s006.docx]

**Supplementary Table 2**: Summary of RNA expression profiling

| Gene ID | Well | AVG ΔC_t_ (Ct(GOI) - Ave Ct (HKG)) | | Fold Change | T-TEST |  |
| --- | --- | --- | --- | --- | --- | --- |
|  |  | Panx1  -/- | Panx1  +/+ | KO/WT | P value |  |
| Adam10 | A01 | 2,79 | 2,79 | 1,00 | 0,981539 |  |
| Adcy1 | A02 | 3,02 | 2,42 | 0,66 | 0,145385 |  |
| Adcy8 | A03 | 8,96 | 8,64 | 0,80 | 0,064365 |  |
| Akt1 | A04 | 3,34 | 3,20 | 0,91 | 0,492683 |  |
| Arc | A05 | 4,90 | 4,27 | 0,64 | 0,139673 |  |
| Bdnf | A06 | 4,20 | 4,35 | 1,11 | 0,500922 |  |
| Camk2a | A07 | -1,87 | -1,98 | 0,93 | 0,534061 |  |
| Camk2g | A08 | 6,07 | 6,02 | 0,97 | 0,943500 |  |
| Cdh2 | A09 | 1,58 | 1,80 | 1,16 | 0,177897 |  |
| Cebpb | A10 | 3,46 | 3,36 | 0,93 | 0,554241 |  |
| Cebpd | A11 | 6,66 | 6,50 | 0,89 | 0,532621 |  |
| Cnr1 | A12 | 1,89 | 2,13 | 1,18 | 0,238474 |  |
| Creb1 | B01 | 6,65 | 6,26 | 0,76 | 0,110006 |  |
| Crem | B02 | 5,22 | 4,97 | 0,84 | 0,367487 |  |
| Dlg4 | B03 | 0,92 | 0,84 | 0,95 | 0,807503 |  |
| Egr1 | B04 | 2,08 | 1,59 | 0,71 | 0,084002 |  |
| Egr2 | B05 | 7,37 | 6,49 | 0,54 | 0,087272 |  |
| Egr3 | B06 | 6,92 | 6,53 | 0,76 | 0,399154 |  |
| Egr4 | B07 | 8,60 | 9,97 | 2,58 | 0,920463 |  |
| Ephb2 | B08 | 5,86 | 6,02 | 1,11 | 0,475310 |  |
| Fos | B09 | 5,35 | 4,73 | 0,65 | 0,180991 |  |
| Gabra5 | B10 | 2,15 | 2,38 | 1,17 | 0,236222 |  |
| Gnai1 | B11 | 1,74 | 2,03 | 1,22 | 0,110233 |  |
| Gria1 | B12 | 0,27 | 0,39 | 1,09 | 0,648490 |  |
| Gria2 | C01 | 0,55 | 0,46 | 0,94 | 0,557502 |  |
| Gria3 | C02 | 2,31 | 1,92 | 0,76 | 0,069639 |  |
| Gria4 | C03 | 4,64 | 4,47 | 0,89 | 0,243831 |  |
| Grin1 | C04 | 3,09 | 2,73 | 0,78 | 0,092342 |  |
| Grin2a | C05 | 2,09 | 2,00 | 0,93 | 0,558431 |  |
| Grin2b | C06 | 2,75 | 2,38 | 0,78 | 0,292084 |  |
| Grin2c | C07 | 8,41 | 7,99 | 0,75 | 0,140596 |  |
| Grin2d | C08 | 5,34 | 5,68 | 1,27 | 0,110103 |  |
| Grip1 | C09 | 5,98 | 6,19 | 1,16 | 0,360889 |  |
| Grm1 | C10 | 5,29 | 5,45 | 1,11 | 0,515089 |  |
| Grm2 | C11 | 4,50 | 4,51 | 1,00 | 0,948603 |  |
| Grm3 | C12 | 3,10 | 3,13 | 1,02 | 0,964007 |  |
| Grm4 | D01 | 6,10 | 6,96 | 1,82 | 0,006330 |  |
| Grm5 | D02 | 2,67 | 2,77 | 1,07 | 0,749853 |  |
| Grm7 | D03 | 3,97 | 3,95 | 0,99 | 0,954939 |  |
| Grm8 | D04 | 6,55 | 6,85 | 1,24 | 0,158499 |  |
| Homer1 | D05 | 2,27 | 2,16 | 0,93 | 0,522744 |  |
| Igf1 | D06 | 6,03 | 6,25 | 1,16 | 0,406178 |  |
| Inhba | D07 | 6,13 | 6,22 | 1,06 | 0,605022 |  |
| Jun | D08 | 3,84 | 3,91 | 1,05 | 0,679617 |  |
| Junb | D09 | 9,42 | 9,47 | 1,03 | 0,784380 |  |
| Kif17 | D10 | 8,86 | 7,57 | 0,41 | 0,228462 |  |
| Klf10 | D11 | 5,15 | 5,03 | 0,92 | 0,373894 |  |
| Mapk1 | D12 | 0,20 | 0,30 | 1,07 | 0,561077 |  |
| Mmp9 | E01 | 7,97 | 8,40 | 1,35 | 0,137277 |  |
| Ncam1 | E02 | 2,53 | 2,55 | 1,01 | 0,992988 |  |
| Nfkb1 | E03 | 5,06 | 5,29 | 1,17 | 0,308273 |  |
| Nfkbib | E04 | 9,01 | 8,87 | 0,90 | 0,374749 |  |
| Ngf | E05 | 6,06 | 6,07 | 1,01 | 0,961315 |  |
| Ngfr | E06 | 6,70 | 7,57 | 1,83 | 0,019172 |  |
| Nos1 | E07 | 6,43 | 6,30 | 0,91 | 0,715878 |  |
| Nptx2 | E08 | 4,38 | 4,65 | 1,20 | 0,309183 |  |
| Nr4a1 | E09 | 4,53 | 4,10 | 0,74 | 0,133452 |  |
| Ntf3 | E10 | 4,84 | 4,29 | 0,68 | 0,071125 |  |
| Ntf5 | E11 | 8,04 | 8,01 | 0,98 | 0,830496 |  |
| Ntrk2 | E12 | 1,28 | 1,36 | 1,05 | 0,686491 |  |
| Pcdh8 | F01 | 1,64 | 1,84 | 1,15 | 0,457562 |  |
| Pick1 | F02 | 3,38 | 3,46 | 1,06 | 0,645507 |  |
| Pim1 | F03 | 6,04 | 5,95 | 0,94 | 0,966372 |  |
| Plat | F04 | 7,41 | 7,62 | 1,16 | 0,551259 |  |
| Plcg1 | F05 | 5,99 | 5,93 | 0,96 | 0,698447 |  |
| Ppp1ca | F06 | 1,33 | 1,39 | 1,04 | 0,732463 |  |
| Ppp1cc | F07 | 0,98 | 0,87 | 0,93 | 0,566947 |  |
| Ppp1r14a | F08 | 4,74 | 4,93 | 1,13 | 0,636026 |  |
| Ppp2ca | F09 | -0,09 | 0,03 | 1,09 | 0,338042 |  |
| Ppp3ca | F10 | -1,67 | -1,54 | 1,09 | 0,415736 |  |
| Prkca | F11 | 0,78 | 0,87 | 1,06 | 0,716892 |  |
| Prkcc | F12 | 0,60 | 0,50 | 0,94 | 0,669401 |  |
| Prkg1 | G01 | 6,44 | 6,37 | 0,95 | 0,706351 |  |
| Rab3a | G02 | -1,72 | -1,57 | 1,11 | 0,229517 |  |
| Rela | G03 | 3,99 | 4,08 | 1,06 | 0,235681 |  |
| Reln | G04 | 2,47 | 2,68 | 1,16 | 0,340968 |  |
| Rgs2 | G05 | 2,88 | 3,04 | 1,12 | 0,318129 |  |
| Rheb | G06 | 1,33 | 1,16 | 0,89 | 0,289688 |  |
| Sirt1 | G07 | 4,27 | 4,28 | 1,01 | 0,942840 |  |
| Srf | G08 | 5,41 | 5,12 | 0,82 | 0,136833 |  |
| Synpo | G09 | 2,12 | 2,14 | 1,01 | 0,994702 |  |
| Timp1 | G10 | 9,24 | 8,91 | 0,80 | 0,386632 |  |
| Tnf | G11 | 12,33 | 11,37 | 0,51 | 0,353180 |  |
| Ywhaq | G12 | 1,10 | 1,27 | 1,12 | 0,177403 |  |
| Gusb | H01 | 4,15 | 4,24 | 1,06 | 0,459315 |  |
| Hprt1 | H02 | 0,70 | 0,72 | 1,01 | 0,816370 |  |
| Hsp90ab1 | H03 | -2,18 | -2,12 | 1,04 | 0,615061 |  |
| Gapdh | H04 | -2,13 | -2,27 | 0,91 | 0,333529 |  |
| Actb | H05 | -0,54 | -0,57 | 0,98 | 0,924151 |  |

**Supplementary Table 2** Summary of data analysis using PCRArrayDataAnalysis_V3.3 software, version August 2010, (http://www.sabiosciences.com/pcrarraydataanalysis.php). Metabotropic glutamate receptor 4 (GRM4) is highlighted in red. Samples in wells H01-H05 were used for normalization of the data set.
