# Supplementary material for: Pannexin1 Stabilizes Synaptic Plasticity and Is Needed for Learning
Source: PLoS One. 2012 Dec 20;7(12):e51767. doi: 10.1371/journal.pone.0051767 (PMC3527502; doi:10.1371/journal.pone.0051767)
Supplement: Methods S1 — This section describes methods used to obtain the data described in the supporting information section. (DOC) [file pone.0051767.s007.doc]

**Supplementary Methods**

**Light microscopy***.* The structural integrity of the brain of Panx1 ^+/+^ and Panx1 ^-/-^ mice (6 to 9 months of age) was assessed after animals were anesthetized with sodium pentobarbital (720 mg/kg Nembutal i.p.) and transcardially perfused with 2.5 % glutaraldehyde in 0.1 M phosphate buffer at pH 7.4 as previously described . Removed brains were documented, embedded in 2% agarose, cut into 1.5 mm coronal brain slices and post fixed with 4% osmium tetroxide. At this stage all slices were photo-documented for later orientation and embedded in Araldite. Slices containing the olfactory bulb, hippocampus and cerebellum were cut into series of semithin sections and stained with 1% toluidine blue. Pictures were taken with an Olympus DP 71 camera mounted on an Olympus microscope BH-2 (Olympus, Münster, Germany), and documented by the computer-assisted analysis system (Soft imaging system GmbH, Münster, Germany).

**Peroxidase immunohistochemistry.** Pannexin 1 ^-/-^ and Panx1 ^+/+^ controls (6 to 9 months of age) were perfused with 4% paraformaldehyde in 0.1 M sodium phosphate buffer (pH 7.4). Blocks with the hippocampus were embedded in agarose as described above, cut into series of 50µm coronal vibratome sections and immunostained as described . For detection of the hippocampal subregions, the polyclonal calbindin antibody (1:10.000; CB38, Swant, Bellinzona, Switzerland) was used. As the activity of parvalbumin-positive interneurons is critical for hippocampal network synchronization, adjacent sections were immunostained with a polyclonal parvalbumin antibody (Supplementary Fig. 4e-h; 1:3000, PV 25; Swant).

**Real Time PCR.** Total RNA was extracted from adult animals of both genotypes (n=4), reverse-transcribed and used in real time PCR as described previously . Each analysis was performed in triplicate and analyzed using Relative Expression Software Tool (REST-2009, ). Primer pairs were:

18s-F, 5’-tgactctttcgaggccctgtaattg-3’,

18s-R, 5’-tggaattaccgcggctgctg-3’;

HSP90-F, 5’-TTTTCCTCCGCGAGTTGATCTCTAATGC-3’,

HSP90-R, 5’-GAGGGTTGGGGATGATGTCAATTTTCAG-3’;

Grm1-F, 5’-ATCGTCAAGCGGTACAACTGGACCTATG-3’,

Grm1-R, 5’-TGCTGTAGATTTTGTCCGAGTGTGCG-3’;

Grm2-F, 5’-cgcctctacaaggacttcgtgctcaatg-3’,

Grm2-R, 5’-cgcagataggtgaagatgttgtagcggc-3’

Grm4-F, 5’-ttgaggaagtggctgaaggtgcagtc-3’,

Grm4-R, 5’-tgtcctcccagaactcagcaaaccag-3’

Grin1-F, 5’-cctgtctcctacacagctggc-3’,

Grin1-R, 5’-acgctggactggtgggagtag-3’

Gria1-F, 5’-ccagatcgatattgtgaacatcag-3’,

Gria1-R, 5’-ccacagaaggaggtcagcatg-3’

**Supplementary Methods References:**

1. Petrasch-Parwez E, Nguyen HP, Lobbecke-Schumacher M, Habbes HW, Wieczorek S, et al. (2007) Cellular and subcellular localization of Huntingtin [corrected] aggregates in the brain of a rat transgenic for Huntington disease. The Journal of comparative neurology 501: 716-730.

2. Kienitz MC, Bender K, Dermietzel R, Pott L, Zoidl G (2011) Pannexin 1 constitutes the large conductance cation channel of cardiac myocytes. The Journal of biological chemistry 286: 290-298.

3. Pfaffl MW, Horgan GW, Dempfle L (2002) Relative expression software tool (REST) for group-wise comparison and statistical analysis of relative expression results in real-time PCR. Nucleic acids research 30: e36.
